# Central visual field sensitivity data from microperimetry with spatially dense sampling

**DOI:** 10.1016/j.dib.2016.07.061

**Published:** 2016-08-04

**Authors:** Andrew T. Astle, Iram Ali, Jonathan Denniss

**Affiliations:** Visual Neuroscience Group, School of Psychology, University of Nottingham, Nottingham NG7 2RD, United Kingdom

**Keywords:** Perimetry, Microperimetry, Visual field, Age-related macular degeneration

## Abstract

Microperimetry, also referred to as fundus perimetry or fundus-driven perimetry, enables simultaneous acquisition of visual sensitivity and eye movement data. We present sensitivity data collected from 60 participants with normal vision using gaze-contingent perimetry. A custom designed spatially dense test grid was used to collect data across the visual field within 13° of fixation. These data are supplemental to a study in which we demonstrated a spatial interpolation method that facilitates comparison of acquired data from any set of spatial locations to normative data and thus screening of individuals with both normal and non-foveal fixation (Denniss and Astle, 2016) [1].

**Specifications Table**

**Table:** 

Subject area	*Medicine, Neuroscience, Psychology*
More specific subject area	*Ophthalmology, Vision Science*
Type of data	*Table and figure*
How data was acquired	*MAIA-2 microperimeter (CenterVue, Padova, Italy)*
Data format	*Formatted*
Experimental factors	*Measurement of visual field sensitivity within 13*° *of fixation in 60 human participants with normal vision using gaze-contingent microperimetry*
Experimental features	*Sensitivity measured using a 4-2 test strategy at 237 test locations spaced 1*° *apart from fixation to an eccentricity of 5*° *and then 2*° *apart out to an eccentricity of 13*°
Data source location	*Nottingham, United Kingdom*
Data accessibility	*Data is within this article*

**Value of the data**

• The presented data provide a detailed baseline for the central region of the hill of vision.
• They allow determination of between-participant variability of luminance increment sensitivity estimates across the central visual field [1].
• The data may be used by those aiming to develop or test by simulation new analysis methods or test procedures for microperimetry.

## 1 Data

**Fig. 1:**
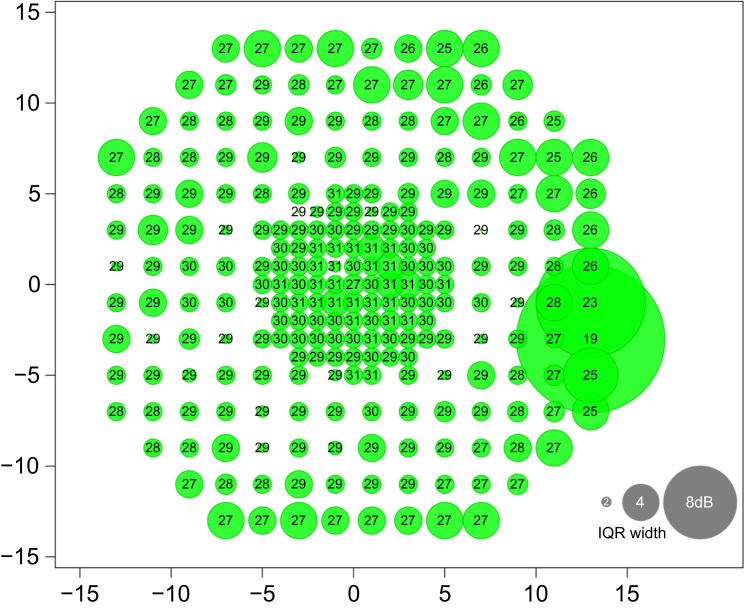
Median and interquartile range width of sensitivity data for all participants at each test location. The number at each location represents median sensitivity (dB). The diameter of the circle at each location represents the interquartile range width of sensitivity thresholds at that test location (dB, see key). Axis notation represents eccentricity in degrees. All data are presented as if they were acquired from right eyes only.

Microperimetry data for 60 healthy participants with normal vision and central (foveal) fixation are provided along separate rows in an annotated .csv file (see online version of this article). Columns contain information on: participant gender, age, eye tested, visual acuity (VA) of the tested eye, sensitivity (in dB) at each test location (specified by *x*,*y* coordinates) and fixation stability data in the form of the mean bivariate contour ellipse area (labeled a–d for the four tests) [2]. A summary of the data can be seen in Fig. 1, which shows the median and variability of sensitivity thresholds at each test location.

## 2 Experimental design, materials and methods

Data were collected from one eye each of 60 healthy participants with no ocular abnormalities and central (foveal) fixation using a MAIA-2 microperimeter (CenterVue, Padova, Italy). All participants were required to be 18 years or older, have spherical refractive error between −15.00 D and +10.00 D, astigmatism of less than 4.00 D, and visual acuity of 0.2 logMAR or better in the tested eye. If both eyes met the inclusion criteria one was randomly selected for testing. All participants completed at least one practice run on a “4-2 Expert” test before the experimental data were collected. All participants provided written informed consent to take part and for their data to be used anonymously in future studies.

Sensitivity was measured with a custom grid of 237 test locations within 13° of fixation using a 4-2 test strategy and Goldmann III (0.43°) stimuli. Test locations were placed on a square grid and spaced 1° apart from fixation to an eccentricity of 5° and then 2° apart to an eccentricity of 13°. Participants were required to fixate the standard 0.76° central annulus target. Data were collected during four randomly ordered test blocks, each containing evenly spaced test locations, with rest periods taken as required. Test blocks with fixation not classified as “stable” by the instrument were discarded and repeated. Due to the influence of the annulus fixation target we advise caution in interpreting the data from the central location (0°, 0°) [3]. In cases where the left eye was tested, data were flipped about the vertical midline such that all data are presented as if they were acquired from right eyes.
