# Supplementary material for: Central visual field sensitivity data from microperimetry with spatially dense sampling
Source: Data Brief. 2016 Aug 4;9:673–5. doi: 10.1016/j.dib.2016.07.061 (PMC5071537; doi:10.1016/j.dib.2016.07.061)
Supplement: Supplementary file 1 — Supplementary material [file mmc1.docx]

**Conflicts of interest:** No conflicting relationship exists for any author

Note, no conflict of interest form available to download from submission website.
